# Supplementary material for: Assessment of Different Niosome Formulations for Optogenetic Applications: Morphological and Electrophysiological Effects
Source: Pharmaceutics. 2023 Jul 1;15(7):1860. doi: 10.3390/pharmaceutics15071860 (PMC10384779; doi:10.3390/pharmaceutics15071860)
Supplement: Supplementary file 1 [file pharmaceutics-15-01860-s001.zip › pharmaceutics-2375563-supplementary.pdf]

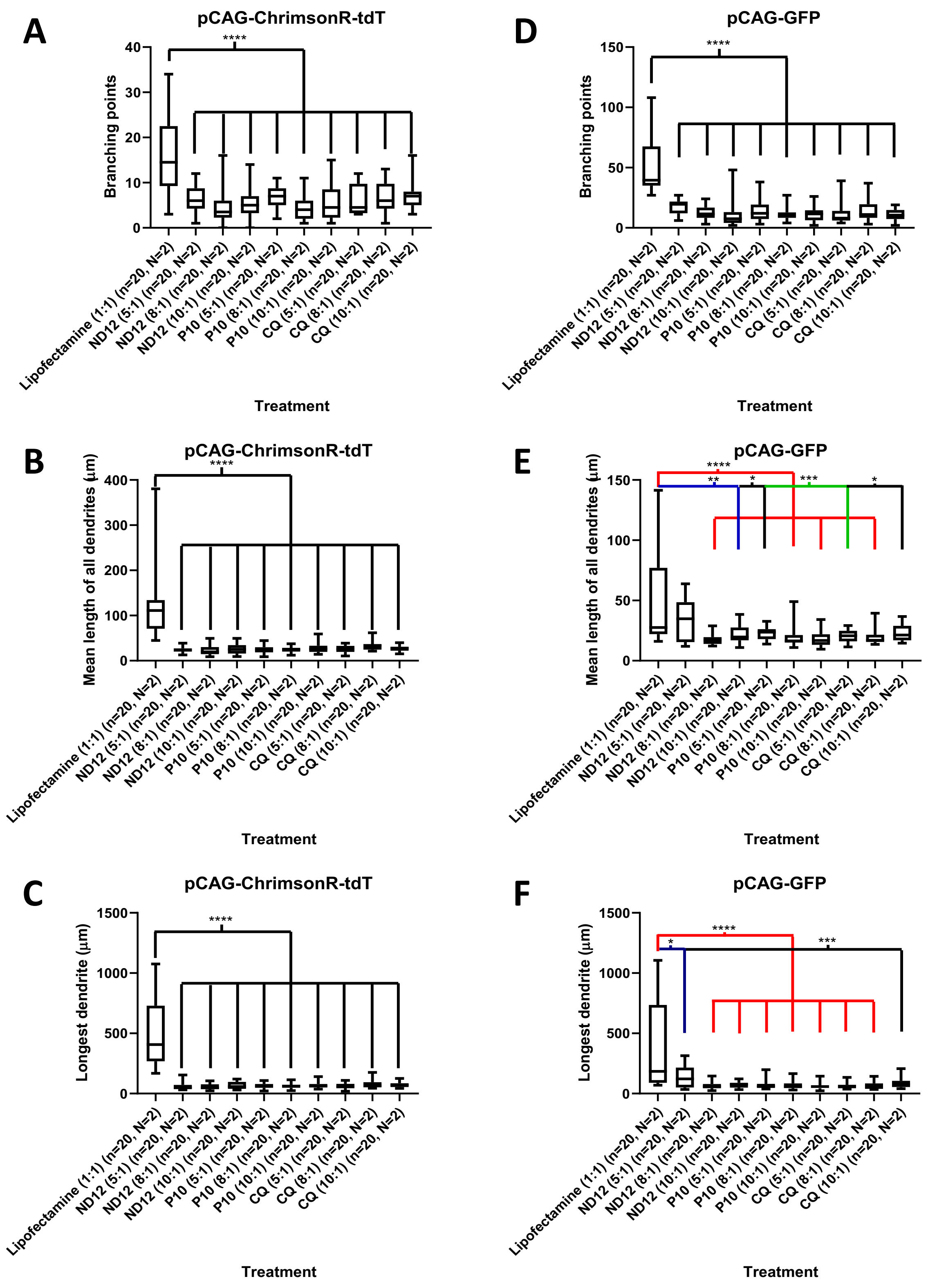

Supplementary Figure S1. Additional morphological parameters in neurons treated with nioplexes made of pCAG-ChrimsonR-tdTomato and pCAG-GFP. 21-28 DIV rat cortical neurons treated with nioplexes made of pCAG-ChrimsonR-dTomato plus niosomes showed reduction in the morphological parameters branching points (A), mean length of all dendrites (B), and longest dendrite (C) compared with the lipofectamine treatment, with similar outcome in neurons treated with nioplexes made of pCAG-GFP plus niosomes (D-F) (Mann-Whitney test, *P<0.05, **P<0.01, ***P<0.001, ****P<0.0001, n = number of cells, N = number of cultures).

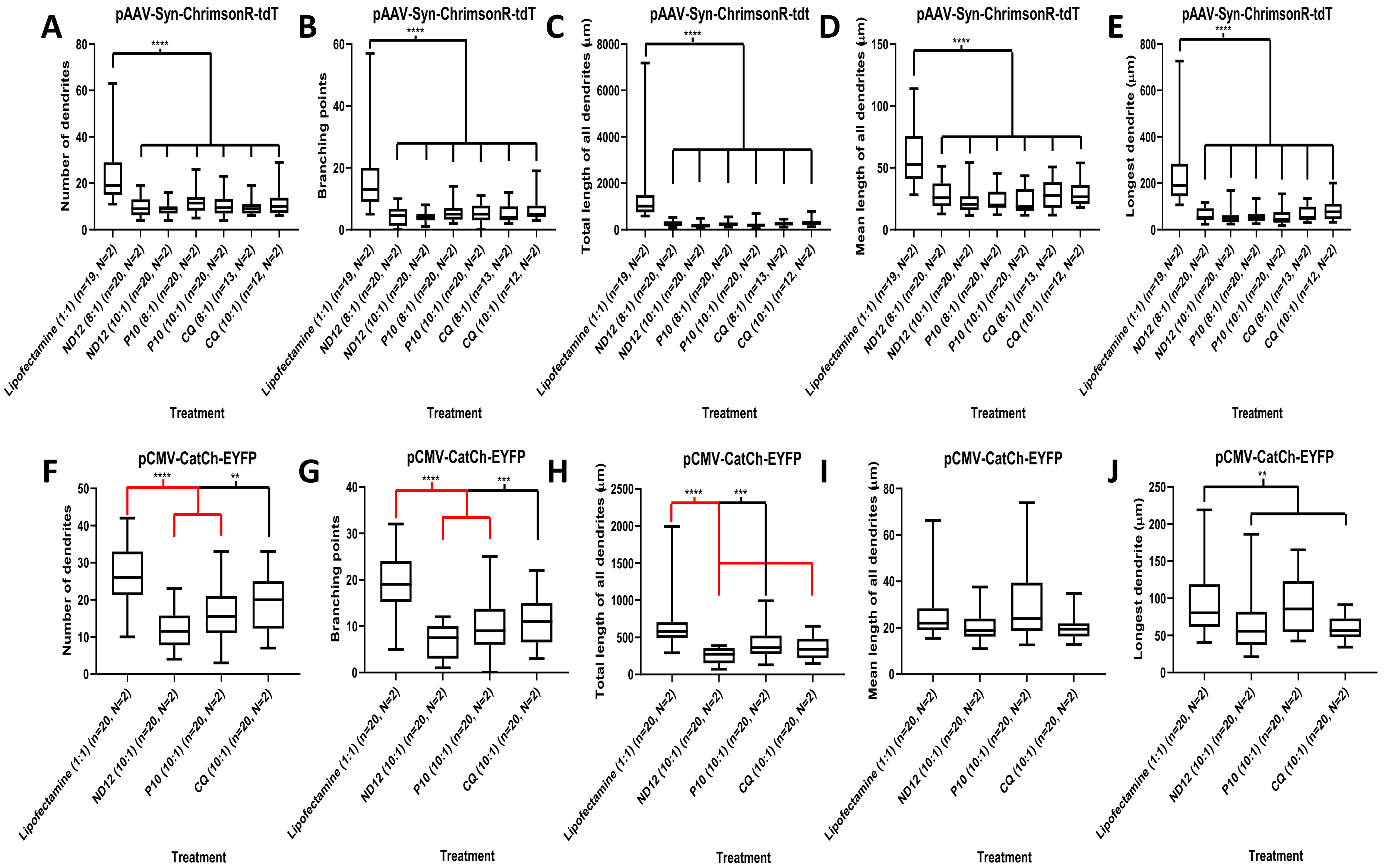

Supplementary Figure S2. Morphological parameters in neurons treated with nioplexes made of pAAV-Syn-ChrimsonR-tdTomato and pCMV-CatCh-EYFP. 21-28 DIV rat cortical neurons treated with nioplexes made of pAAV-Syn-ChrimsonR-tdTomato plus niosomes showed reduction in morphological parameters number of dendrites (A), branching points (B), total length of all dendrites (C), mean length of all dendrites (D) and longest dendrite (E) compared with the lipofectamine treatment, with similar outcome in pCMV-CatCh-EYFP in most parameters (F, G, H. J), except in mean length of all dendrites (I) (Mann-Whitney test, **P<0.01, ***P<0.001, ****P<0.0001, n = number of cells, N = number of cultures).

Nioplexes<br>21-28 DIV neurons	Number of dendrites	Branching points	Total length of all dendrites	Mean length of all dendrites	Longest dendrite
	CAG-ChrimsonR/Lipofectamine (1:1) (n=20, N=2)	CAG-ChrimsonR/Lipofectamine (1:1) (n=20, N=2)	CAG-ChrimsonR/Lipofectamine (1:1) (n=20, N=2)	CAG-ChrimsonR/Lipofectamine (1:1) (n=20, N=2)	CAG-ChrimsonR/Lipofectamine (1:1) (n=20, N=2)
CAG-ChrimsonR/ND12 (5:1) (n=20, N=2)	p<0.0001****	p<0.0001****	p<0.0001****	p<0.0001****	p<0.0001****
CAG-ChrimsonR/ND12 (8:1) (n=20, N=2)	p<0.0001****	p<0.0001****	p<0.0001****	p<0.0001****	p<0.0001****
CAG-ChrimsonR/ND12 (10:1) (n=20, N=2)	p=0.0002***	p<0.0001****	p<0.0001****	p<0.0001****	p<0.0001****
CAG-ChrimsonR/P10 (5:1) (n=20, N=2)	p=0.0001***	p<0.0001****	p<0.0001****	p<0.0001****	p<0.0001****
CAG-ChrimsonR/P10 (8:1) (n=20, N=2)	p<0.0001****	p<0.0001****	p<0.0001****	p<0.0001****	p<0.0001****
CAG-ChrimsonR/P10 (10:1) (n=20, N=2)	p<0.0001****	p<0.0001****	p<0.0001****	p<0.0001****	p<0.0001****
CAG-ChrimsonR/CQ (5:1) (n=20, N=2)	p<0.0001****	p<0.0001****	p<0.0001****	p<0.0001****	p<0.0001****
CAG-ChrimsonR/CQ (8:1) (n=20, N=2)	p<0.0001****	p<0.0001****	p<0.0001****	p<0.0001****	p<0.0001****
CAG-ChrimsonR/CQ (10:1) (n=20, N=2)	p=0.0001***	p<0.0001****	p<0.0001****	p<0.0001****	p<0.0001****
	CatCh/Lipofectamine (1:1) (n=20, N=2)	CatCh/Lipofectamine (1:1) (n=20, N=2)	CatCh/Lipofectamine (1:1) (n=20, N=2)	CatCh/Lipofectamine (1:1) (n=20, N=2)	CatCh/Lipofectamine (1:1) (n=20, N=2)
CatCh /ND12 (10:1) (n=20, N=2)	p<0.0001****	p<0.0001****	p<0.0001****	p=0.1207	p=0.0087**
CatCh /P10 (10:1) (n=20, N=2)	p<0.0001****	p<0.0001****	p=0.0008***	p=0.8620	p=0.7180
CatCh /CQ (10:1) (n=20, N=2)	p=0.0065**	p=0.0002***	p<0.0001****	p=0.0675	p=0.0012**
	Syn-ChrimsonR/Lipofectamine (1:1) (n=19, N=2)	Syn-ChrimsonR/Lipofectamine (1:1) (n=19, N=2)	Syn-ChrimsonR/Lipofectamine (1:1) (n=19, N=2)	Syn-ChrimsonR/Lipofectamine (1:1) (n=19, N=2)	Syn-ChrimsonR/Lipofectamine (1:1) (n=19, N=2)
Syn-ChrimsonR /ND12 (8:1) (n=20, N=2)	p<0.0001****	p<0.0001****	p<0.0001****	p<0.0001****	p<0.0001****
Syn-ChrimsonR /ND12 (10:1) (n=20, N=2)	p<0.0001****	p<0.0001****	p<0.0001****	p<0.0001****	p<0.0001****
Syn-ChrimsonR /P10 (8:1) (n=20, N=2)	p<0.0001****	p<0.0001****	p<0.0001****	p<0.0001****	p<0.0001****
Syn-ChrimsonR /P10 (10:1) (n=20, N=2)	p<0.0001****	p<0.0001****	p<0.0001****	p<0.0001****	p<0.0001****
Syn-ChrimsonR /CQ (8:1) (n=13, N=2)	p<0.0001****	p<0.0001****	p<0.0001****	p<0.0001****	p<0.0001****
Syn-ChrimsonR /CQ (10:1) (n=12, N=2)	p<0.0001****	p<0.0001****	p<0.0001****	p<0.0001****	p<0.0001****
	GFP/Lipofectamine (1:1) (n=20, N=2)	GFP/Lipofectamine (1:1) (n=20, N=2)	GFP/Lipofectamine (1:1) (n=20, N=2)	GFP/Lipofectamine (1:1) (n=20, N=2)	GFP/Lipofectamine (1:1) (n=20, N=2)
GFP/ND12 (5:1) (n=20, N=2)	p<0.0001****	p<0.0001****	p<0.0001****	p=0.1653	p=0.0283*
GFP/ND12 (8:1) (n=20, N=2)	p<0.0001****	p<0.0001****	p<0.0001****	p<0.0001****	p<0.0001****
GFP/ND12 (10:1) (n=20, N=2)	p<0.0001****	p<0.0001****	p<0.0001****	p=0.0019**	p<0.0001****
GFP/P10 (5:1) (n=20, N=2)	p<0.0001****	p<0.0001****	p<0.0001****	p=0.0103*	p<0.0001****
GFP/P10 (8:1) (n=20, N=2)	p<0.0001****	p<0.0001****	p<0.0001****	p<0.0001****	p<0.0001****
GFP/P10 (10:1) (n=20, N=2)	p<0.0001****	p<0.0001****	p<0.0001****	p<0.0001****	p<0.0001****
GFP/CQ (5:1) (n=20, N=2)	p<0.0001****	p<0.0001****	p<0.0001****	p=0.0007***	p<0.0001****
GFP/CQ (8:1) (n=20, N=2)	p<0.0001****	p<0.0001****	p<0.0001****	p<0.0001****	p<0.0001****
GFP/CQ (10:1) (n=20, N=2)	p<0.0001****	p<0.0001****	p<0.0001****	p=0.0155*	p=0.0002***

Supplementary Table S1. p-values of 21-28 DIV neurons treated with nioplexes compared with lipofectamine treated neurons in morphological analysis.

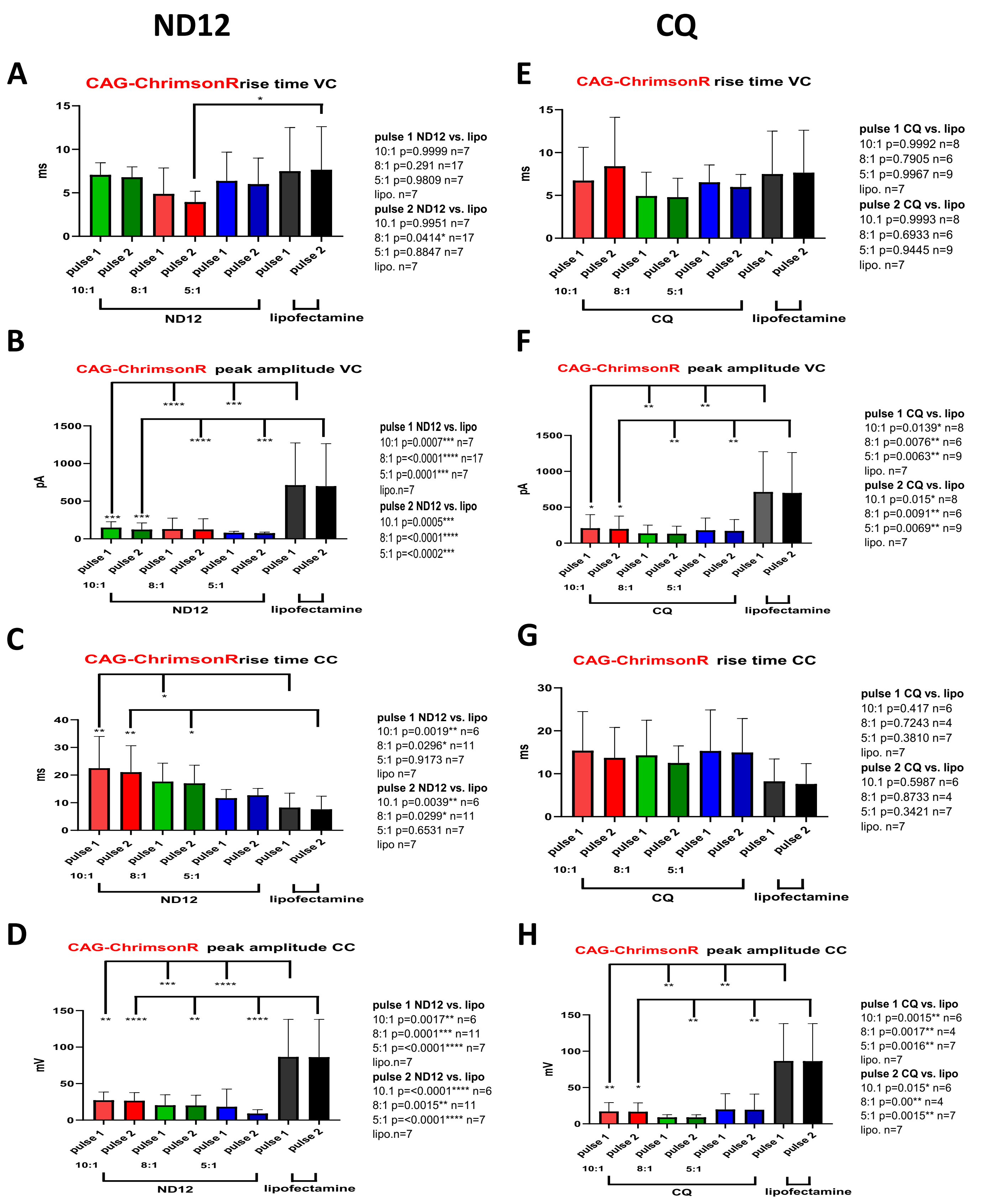

Supplementary Figure S3. Additional electrophysiological changes produced by nioplexes with CAG-ChrimsonR in rat cortical neurons. Photostimulation of rat cortical neuron DIV 28 expressing CAG-ChrimsonR transfected with niosomes or lipofectamine (1:1) with two pulses of 5 ms (590 nm) with a 1-second interspace. (A-D) Comparison between lipofectamine and ND12 niosomes at different ratios in rise time and peak amplitude electrophysiological parameters in each light pulse in VC and CC modes, showing reduced peak amplitudes in neurons treated with niosomes and, in some cases, increased rise rimes. (E-H) Comparison between lipofectamine and CQ niosomes at different ratios in rise time and peak amplitude electrophysiological parameters in each light pulse in VC and CC modes, showing reduced peak amplitudes in neurons treated with niosomes, but no differences in rise time (ordinary one-way ANOVA, *P<0.05, **P<0.01, ***P<0.001, ****P<0.0001). Graphs bars are expressed as mean ± SD.

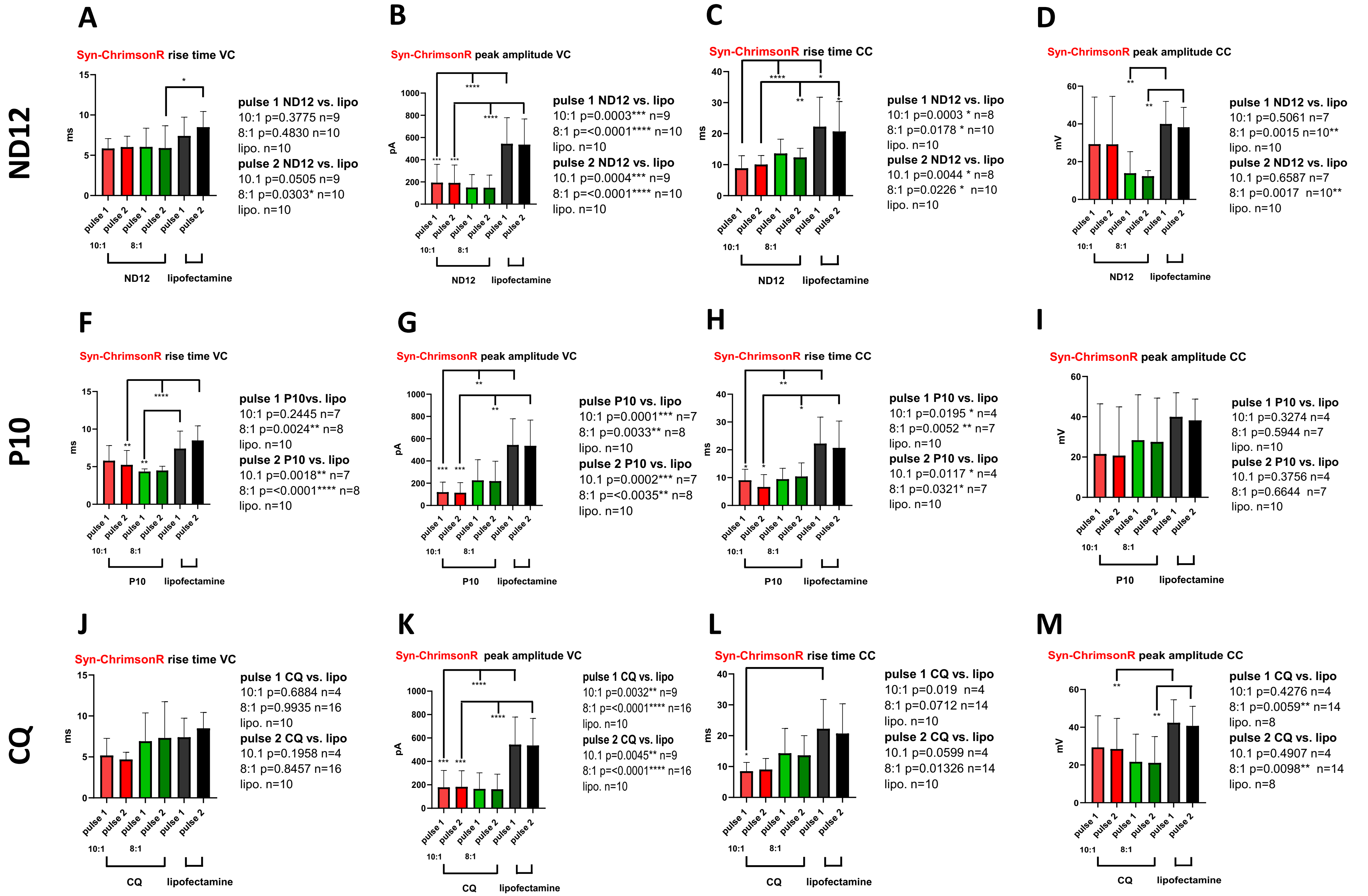

Supplementary Figure S4. Electrophysiological changes produced by nioplexes with Syn-ChrimsonR in rat cortical neurons. Photostimulation of rat cortical neuron DIV 28 expressing Syn-ChrimsonR transfected with niosomes or lipofectamine (1:1) with two pulses of 5 ms (590 nm) with a 1-second interspace. (A-D) Comparison between lipofectamine and ND12, niosomes (F-I) lipofectamine and P10 niosomes, and (J-M) lipofectamine and CQ niosomes at different ratios in rise time and peak amplitude electrophysiological parameters in each light pulse in VC and CC modes, showing reduced peak amplitudes in neurons treated with niosomes (although not in all cases, as it happens in CC peak amplitude with P10 niosomes) and, in some cases, reduced rise rimes (ordinary one-way ANOVA, *P<0.05, **P<0.01, ***P<0.001, ****P<0.0001). Graphs bars are expressed as mean ± SD.

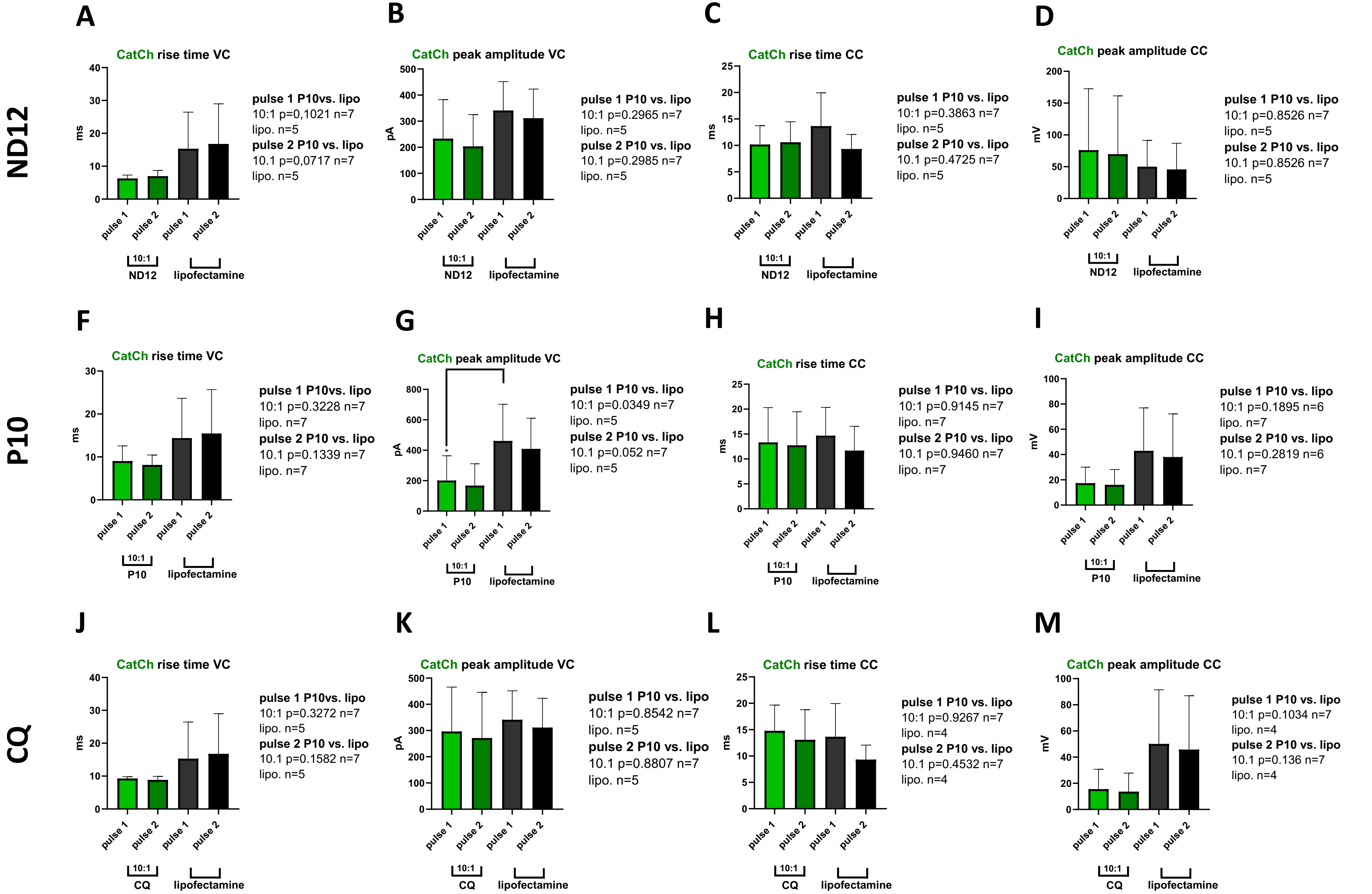

Supplementary Figure S5. Electrophysiological changes produced by nioplexes with CatCh in rat cortical neurons. Photostimulation of rat cortical neuron DIV 21-28 expressing CatCh transfected with niosomes or lipofectamine (1:1) with two pulses of 5 ms (590 nm) with a 1-second interspace. (A-D) Comparison between lipofectamine and ND12, niosomes (F-I) lipofectamine and P10 niosomes, and (J-M) lipofectamine and CQ niosomes at different ratios in rise time and peak amplitude electrophysiological parameters in each light pulse in VC and CC modes, showing no statistical difference in any parameter, except in VC peak amplitude in P10 niosomes (ordinary one-way ANOVA, *P<0.05, **P<0.01, ***P<0.001, ****P<0.0001). Graphs bars are expressed as mean ± SD.

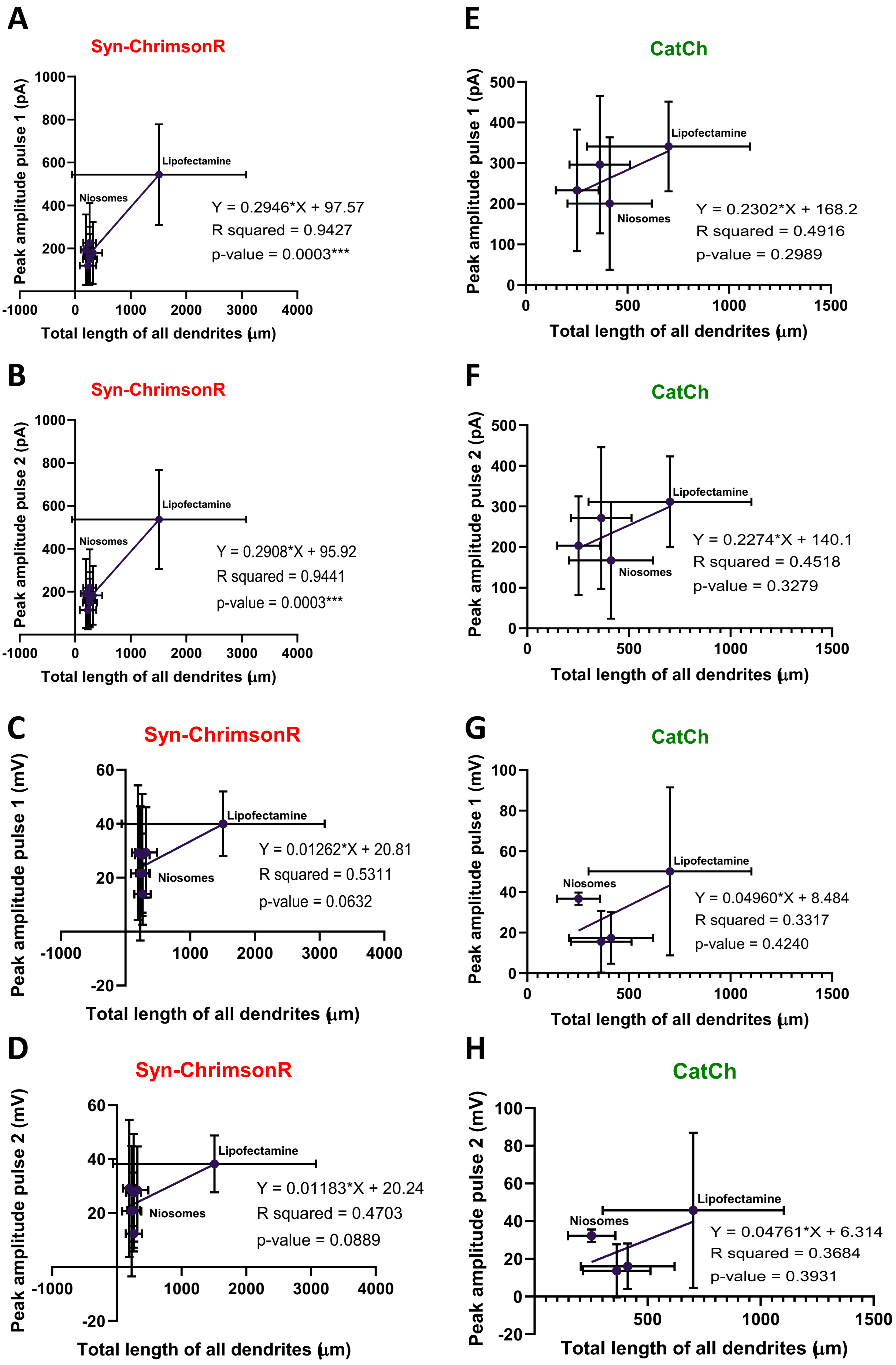

Supplementary Figure S6. Morphological and electrophysiological correlations with Syn-ChrimsonR and CatCh plasmids. (A-D) Dispersion graphs correlating total length of all dendrites morphological parameter with peak amplitude electrophysiological parameters in Syn-ChrimsonR expressing neurons, existing positive correlationship and statistical significance with VC recordings in pulses 1 (A) and 2 (B), but not in CC recordings in pulses 1 (C) and 2 (D). (E-H) Dispersion graphs correlating total length of all dendrites morphological parameter with peak amplitude electrophysiological parameters in CatCh expressing neurons, with weak positive correlation and no statistical significance in both VC (E-F) and CC (G-H) recordings (simple linear regression, ***$P<0.001$). Dots represent mean, horizontal graphs morphological (X axis) SD and vertical graphs electrophysiological (Y axis) SD.

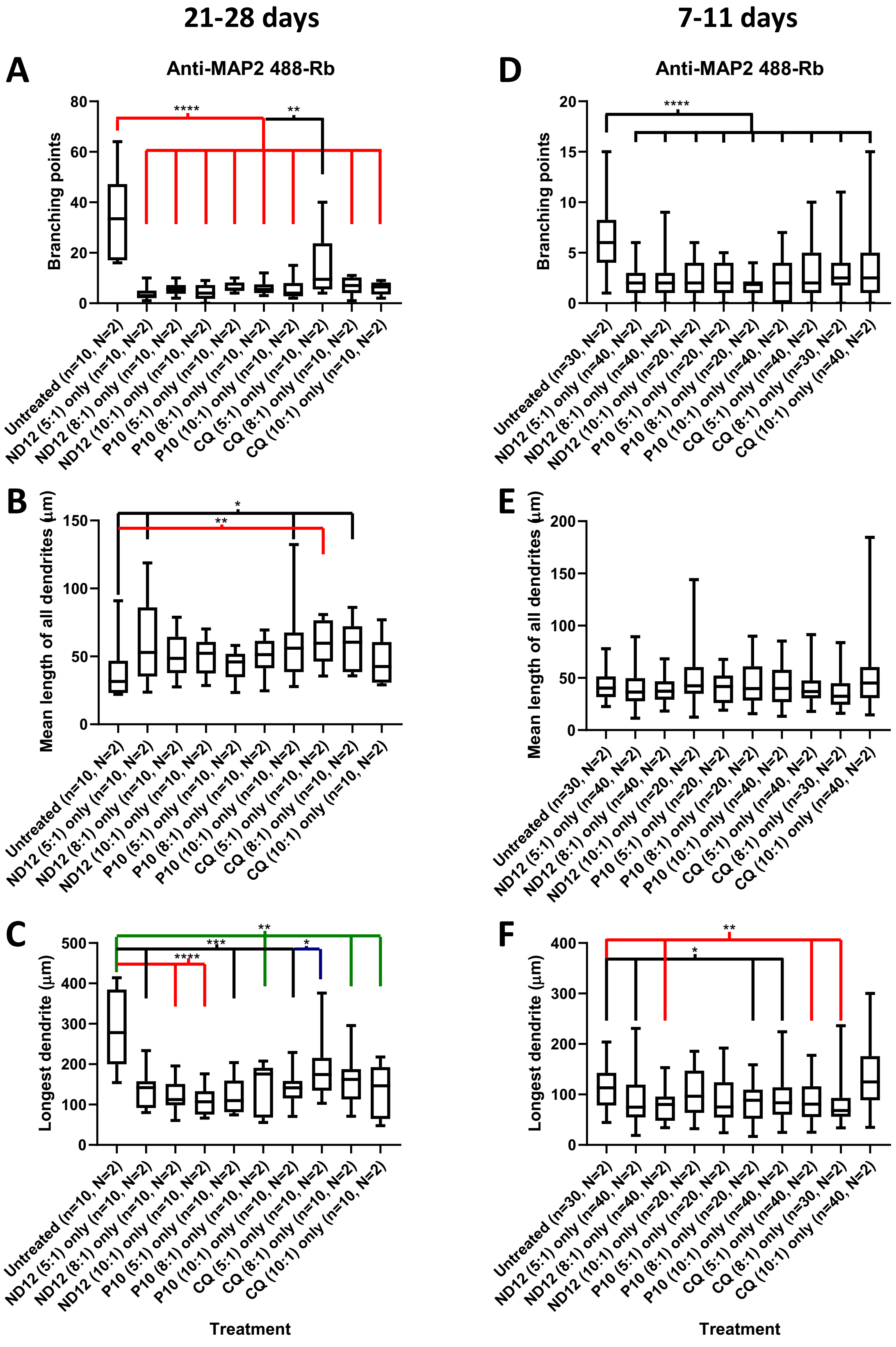

Supplementary Figure S7. Additional morphological changes induced by niosomes alone in both 21-28 DIV and 7-11 DIV rat cortical neurons. Both 21-28 DIV and 7-11 DIV rat cortical neurons treated with only niosomes showed several morphological alterations in their branching points (Mann-Whitney test, *P<0.05, **P<0.01, ***P<0.001, ****P<0.0001, n = number of cells, N = number of cultures).

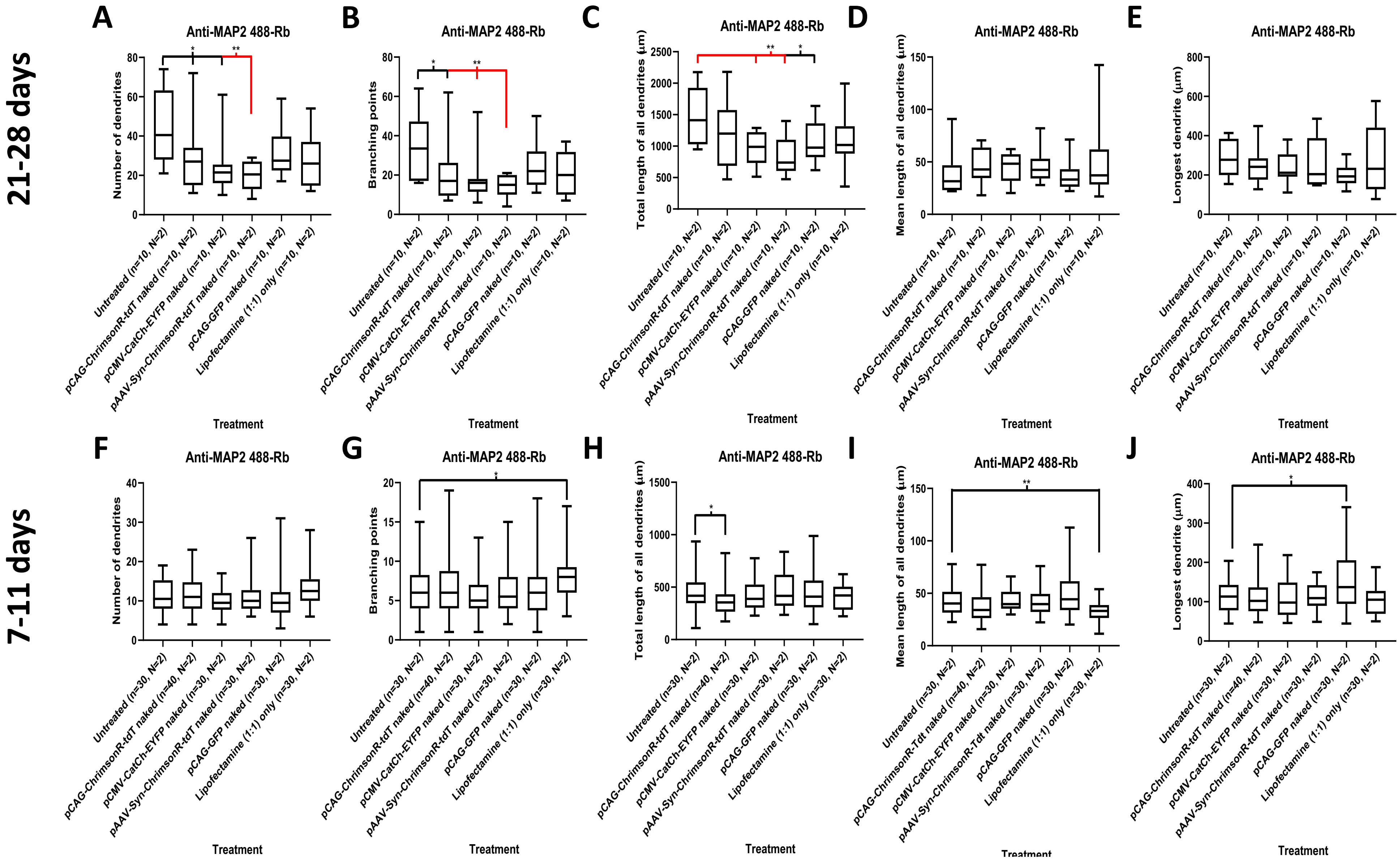

Supplementary Figure S8. Morphological parameters in neurons treated with naked plasmids and only lipofectamine in both 21-28 DIV and 7-11 DIV rat cortical neurons. 21-28 DIV rat cortical neurons treated with naked plasmids showed, depending on the plasmid used, some reductions in the morphological parameters number of dendrites (A), branching points (B), and total length of all dendrites (C)(, while the morphological parameters mean length of all dendrites (D) and longest dendrite (E) remained unaffected. On the other hand, 7-11 DIV neurons showed no statistical significance among treatments in number of dendrites (F), but they did in some cases in branching points (G), total length of all dendrites (H), mean length of all dendrites (I), and longest dendrite (J) (Mann-Whitney test, *P<0.05, **P<0.01, n = number of cells, N = number of cultures).

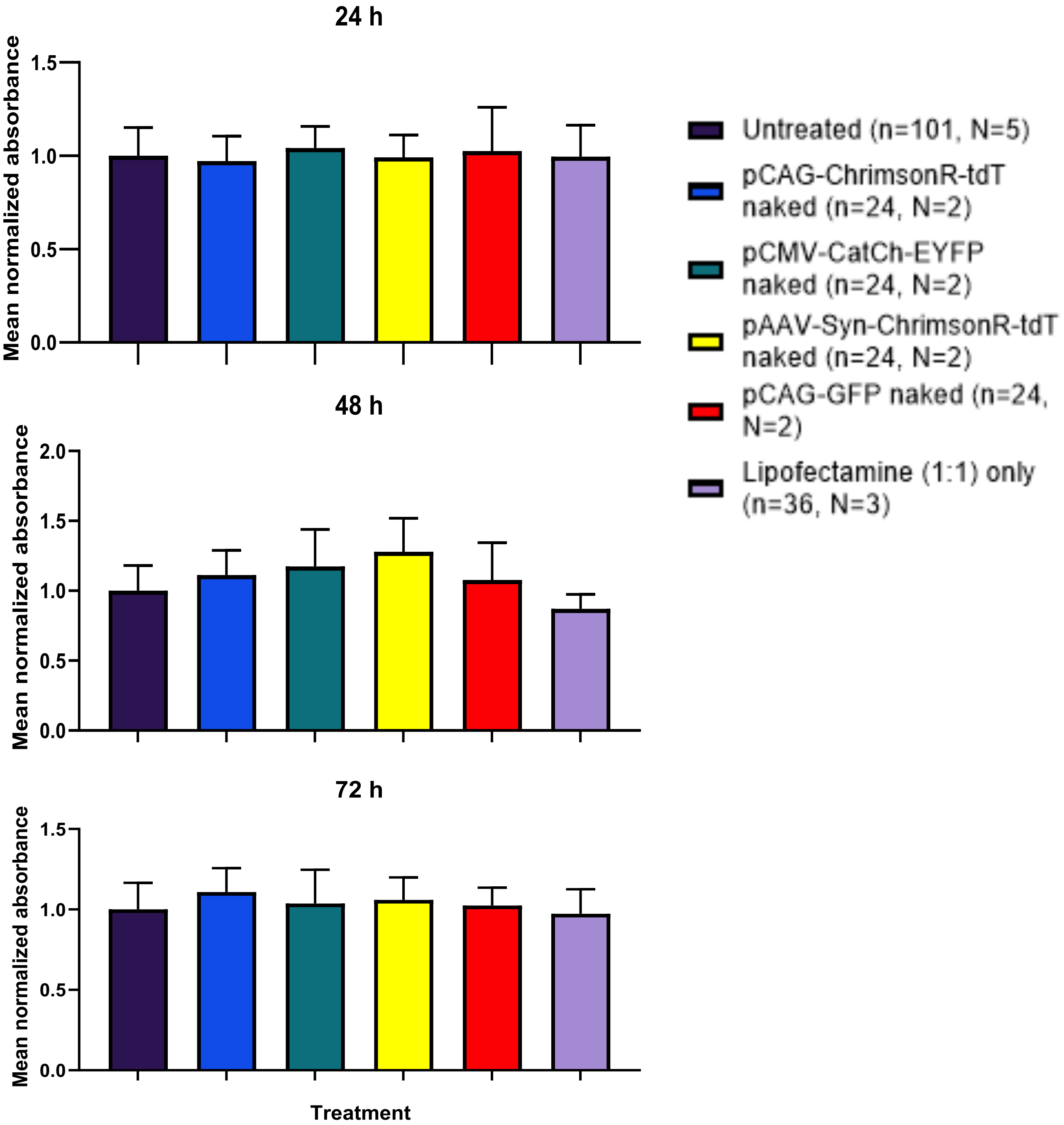

Supplementary Figure S9. Cell viability with naked plasmids and lipofectamine is not affected in 21-28 DIV cortical neurons. MTT assays performed at 24, 48 and 72 h in 21-28 DIV rat cortical neurons showed no reduction in cell viability with naked plasmids nor lipofectamine only compared with untreated neurons. (Multiple t-tests, *P<0.05, ***P<0.001, ****P<0.0001, n = number of wells, N = number of cultures). Graph bars are expressed as mean ± SD.

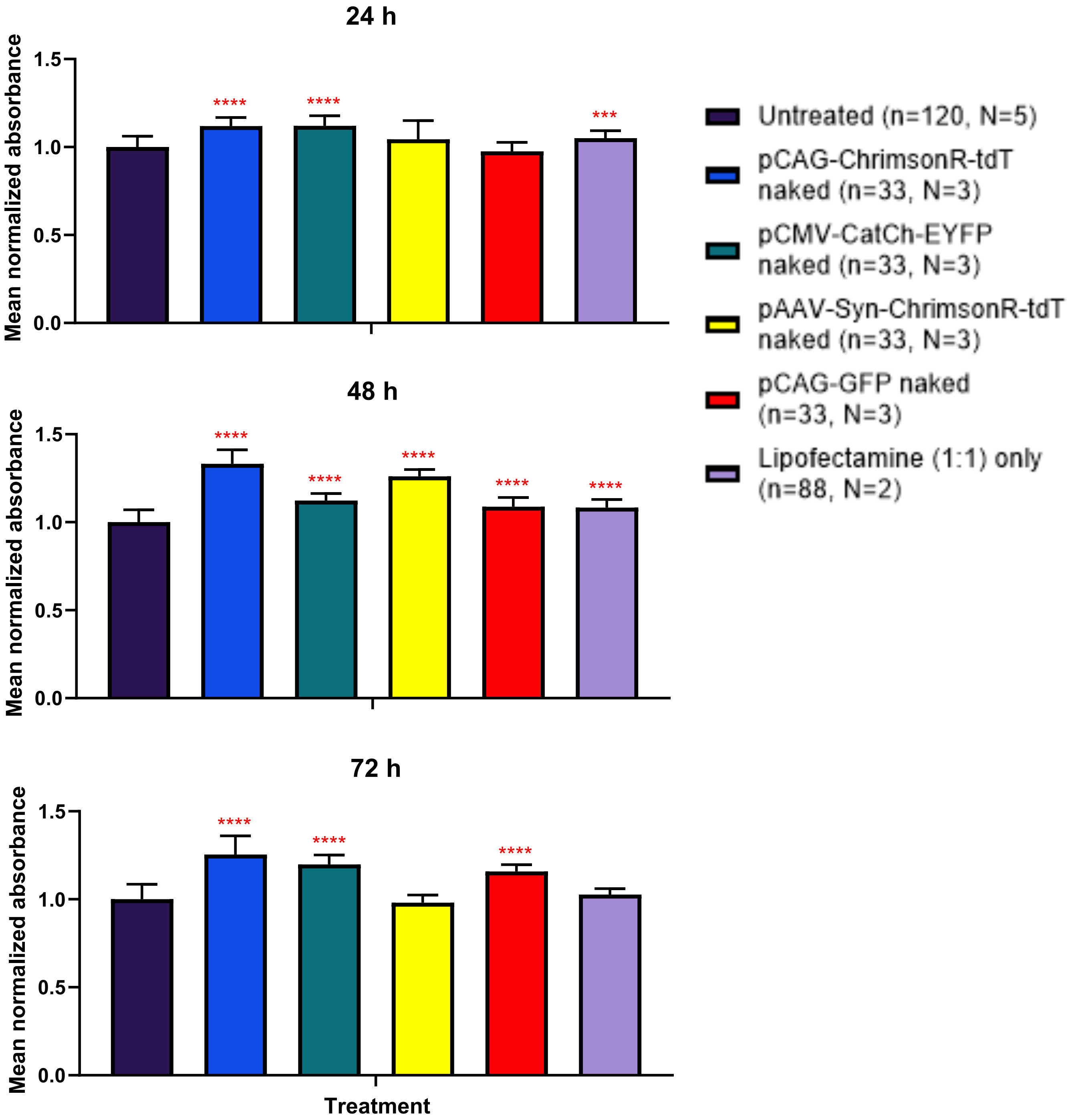

Supplementary Figure S10. Cell viability with naked plasmids and lipofectamine is not affected in 7-11 DIV cortical neurons. MTT assays performed at 24, 48 and 72 h in 7-11 DIV rat cortical neurons showed no reduction in cell viability with naked plasmids nor lipofectamine only compared with untreated neurons. (Multiple t-tests, **P*<0.05, ****P*<0.001, *****P*<0.0001, n = number of wells, N = number of cultures). Red p-values mean that there is statistical differen ce with groups with higher mean values than the untreated groups. Graph bars are expressed as mean ± SD.

Anti-MAP2 488-Rb 21-28 days neurons	Number of dendrites	Branching points	Total length of all dendrites	Mean length of all dendrites	Longest dendrite
	Untreated (n=10, N=2)	Untreated (n=10, N=2)	Untreated (n=10, N=2)	Untreated (n=10, N=2)	Untreated (n=10, N=2)
ND12 (5:1) only (n=10, N=2)	p<0.0001****	p<0.0001****	p<0.0001****	p=0.0355*	p=0.0002***
ND12 (8:1) only (n=10, N=2)	p<0.0001****	p<0.0001****	p<0.0001****	p=0.0753	p<0.0001****
ND12 (10:1) only (n=10, N=2)	p<0.0001****	p<0.0001****	p<0.0001****	p=0.0630	p<0.0001****
P10 (5:1) only (n=10, N=2)	p<0.0001****	p<0.0001****	p<0.0001****	p=0.1431	p=0.0001***
P10 (8:1) only (n=10, N=2)	p<0.0001****	p<0.0001****	p=0.0002***	p=0.0630	p=0.0029**
P10 (10:1) only (n=10, N=2)	p<0.0001****	p<0.0001****	p<0.0001****	p=0.0288*	p=0.0002***
CQ (5:1) only (n=10, N=2)	p=0.0042**	p=0.0041**	p=0.1051	p=0.0089**	p=0.0232*
CQ (8:1) only (n=10, N=2)	p<0.0001****	p<0.0001****	p=0.0003***	p=0.0147*	p=0.0039**
CQ (10:1) only (n=10, N=2)	p<0.0001****	p<0.0001****	p<0.0001****	p=0.1655	p=0.0011**
CAG-ChrimsonR naked (n=10, N=2)	p=0.0498*	p=0.0410*	p=0.2799	p=0.2176	p=0.3527
CatCh naked (n=10, N=2)	p=0.0136*	p=0.0081**	p=0.0068**	p=0.1903	p=0.2475
Syn-ChrimsonR naked (n=10, N=2)	p=0.0014**	p=0.0023**	p=0.0039**	p=0.1279	p=0.3930
GFP naked (n=10, N=2)	p=0.1593	p=0.1175	p=0.0232*	p=0.7959	p=0.0524
Lipofectamine (1:1) only (n=10, N=2)	p=0.0502	p=0.0658	p=0.1051	p=0.4359	p=0.5787

Supplementary Table S2. p-values of 21-28 DIV neurons treated with only niosomes or naked plasmids compared with untreated neurons in morphological analysis.

MTT 21-28 days neurons	Untreated (n=101, N=5)		
	24 h	48 h	72h
ND12 (5:1) only (n=24, N=2)	p=0.6033	p=0.9098	p=0.9098
ND12 (8:1) only (n=42, N=2)	p<0.0001****	p=0.1231	p=0.4691
ND12 (10:1) only (n=41, N=4)	p<0.0001****	p=0.0367*	p=0.0251*
P10 (5:1) only (n=24, N=2)	p=0.9571	p=0.1080	p=0.7230
P10 (8:1) only (n=43, N=4)	p=0.0309*	p=0.2647	p=0.0022**
P10 (10:1) only (n=42, N=4)	p=0.0383*	p=0.0198*	p=0.9059
CQ (5:1) only (n=24, N=2)	p=0.2889	p=0.6243	p=0.5354
CQ (8:1) only (n=43, N=4)	p=0.0001***	p<0.0001****	p=0.1055
CQ (10:1) only (n=42, N=4)	p<0.0001****	p=0.0011**	p=0.5538
CAG-ChrimsonR naked (n=24, N=2)	p=0.6213	p=0.2783	p=0.2783
CatCh naked (n=24, N=2)	p=0.7266	p=0.0840	p=0.7266
Syn-ChrimsonR naked (n=24, N=2)	p=0.8775	p=0.0017**	p=0.5912
GFP naked (n=24, N=2)	p=0.9125	p=0.6969	p=0.9125
Lipofectamine (1:1) only (n=36, N=3)	p=0.9319	p=0.0720	p=0.8620

Supplementary Table S3. p-values of 21-28 DIV neurons treated with only niosomes or naked plasmids compared with untreated neurons in MTT assays.

MTT Nioplexes<br>21-28 DIV neurons			
8:1 proportion		10:1 proportion	
	Untreated (n=29, N=2)		Untreated (n=30, N=2)
CAG-ChrimsonR/ND12 (8:1) (n=10, N=2)	p=0.0070**	CAG-ChrimsonR/ND12 (10:1) (n=10, N=2)	p<0.0001****
CAG-ChrimsonR/P10 (8:1) (n=10, N=2)	p=0.9233	CAG-ChrimsonR/P10 (10:1) (n=10, N=2)	p=0.0129*
CAG-ChrimsonR/CQ (8:1) (n=10, N=2)	p<0.0001****	CAG-ChrimsonR/CQ (10:1) (n=10, N=2)	p=0.0002***
CatCh /ND12 (8:1) (n=12, N=2)	p=0.0010**	CatCh /ND12 (10:1) (n=10, N=2)	p=0.0002***
CatCh /P10 (8:1) (n=12, N=2)	p=0.1561	CatCh /P10 (10:1) (n=10, N=2)	p=0.1531
CatCh /CQ (8:1) (n=12, N=2)	p<0.0001****	CatCh /CQ (10:1) (n=10, N=2)	p=0.0002***
Syn-ChrimsonR /ND12 (8:1) (n=11, N=2)	p<0.0001****	Syn-ChrimsonR /ND12 (10:1) (n=10, N=2)	p=0.0010**
Syn-ChrimsonR /P10 (8:1) (n=11, N=2)	p=0.1027	Syn-ChrimsonR /P10 (10:1) (n=10, N=2)	p=0.0002***
Syn-ChrimsonR /CQ (8:1) (n=11, N=2)	p=0.0002***	Syn-ChrimsonR /CQ (10:1) (n=10, N=2)	p<0.0001****
GFP/ND12 (8:1) (n=10, N=2)	p=0.0204*	GFP/ND12 (10:1) (n=10, N=2)	p=0.0017**
GFP/P10 (8:1) (n=10, N=2)	p=0.1807	GFP/P10 (10:1) (n=10, N=2)	p=0.7845
GFP/CQ (8:1) (n=10, N=2)	p=0.0663	GFP/CQ (10:1) (n=10, N=2)	p<0.0001****

Supplementary Table S4. p-values of 21-28 DIV neurons treated with nioplexes compared with untreated neurons in MTT assays.

Anti-MAP2 488-Rb<br>7-11 days neurons	Number of dendrites	Branching points	Total length of all dendrites	Mean length of all dendrites	Longest dendrite
	Untreated (n=30, N=2)	Untreated (n=30, N=2)	Untreated (n=30, N=2)	Untreated (n=30, N=2)	Untreated (n=30, N=2)
ND12 (5:1) only (n=40, N=2)	p<0.0001****	p<0.0001****	p<0.0001****	p=0.6561	p=0.0210*
ND12 (8:1) only (n=40, N=2)	p<0.0001****	p<0.0001****	p<0.0001****	p=0.2601	p=0.0013**
ND12 (10:1) only (n=20, N=2)	p<0.0001****	p<0.0001****	p<0.0001****	p=0.2755	p=0.3678
P10 (5:1) only (n=20, N=2)	p<0.0001****	p<0.0001****	p<0.0001****	p=0.8791	p=0.0697
P10 (8:1) only (n=20, N=2)	p<0.0001****	p<0.0001****	p<0.0001****	p=0.9101	p=0.0481*
P10 (10:1) only (n=40, N=2)	p<0.0001****	p<0.0001****	p<0.0001****	p=0.9084	p=0.0262*
CQ (5:1) only (n=40, N=2)	p<0.0001****	p<0.0001****	p<0.0001****	p=0.4238	p=0.0077**
CQ (8:1) only (n=30, N=2)	p<0.0001****	p<0.0001****	p<0.0001****	p=0.0627	p=0.0017**
CQ (10:1) only (n=40, N=2)	p=0.0001***	p<0.0001****	p<0.0001****	p=0.3053	p=0.1571
CAG-ChrimsonR naked (n=40, N=2)	p=0.8291	p=0.8611	p=0.0356*	p=0.1142	p=0.6182
CatCh naked (n=30, N=2)	p=0.2296	p=0.3330	p=0.4492	p=0.5204	p=0.6973
Syn-ChrimsonR naked (n=30, N=2)	p=0.8707	p=0.9853	p=0.9707	p=0.8172	p=0.9474
GFP naked (n=30, N=2)	p=0.3205	p=0.6766	p=0.7302	p=0.291	p=0.0199*
Lipofectamine (1:1) only (n=30, N=2)	p=0.1479	p=0.0122*	p=0.4761	p=0.0054**	p=0.3659

Supplementary Table S5. p-values of 7-11 DIV neurons treated with only niosomes or naked plasmids compared with untreated neurons in morphological analysis.

MTT<br>7-11 DIV neurons	Untreated (n=120, N=5)		
	24 h	48 h	72h
ND12 (5:1) only (n=32, N=4)	p=0.009**	p=0.0005***	p=0.5887
ND12 (8:1) only (n=33, N=4)	p<0.0001****	p=0.0001***	p=0.0040**
ND12 (10:1) only (n=32, N=4)	p<0.0001****	p<0.0001****	p<0.0001****
P10 (5:1) only (n=32, N=4)	p=0.0002***	p=0.0683	p=0.5073
P10 (8:1) only (n=33, N=4)	p=0.0018**	p<0.0001****	p<0.0001****
P10 (10:1) only (n=33, N=4)	p<0.0001****	p<0.0001****	p<0.0001****
CQ (5:1) only (n=33, N=4)	p<0.0001****	p<0.0001****	p=0.4979
CQ (8:1) only (n=33, N=4)	p<0.0001****	p<0.0001****	p<0.0001****
CQ (10:1) only (n=33, N=4)	p<0.0001****	p<0.0001****	p<0.0001****
CAG-ChrimsonR naked (n=33, N=4)	p<0.0001****	p<0.0001****	p<0.0001****
CatCh naked (n=33, N=4)	p<0.0001****	p<0.0001****	p<0.0001****
Syn-ChrimsonR naked (n=33, N=4)	p=0.1510	p<0.0001****	p=0.4697
GFP naked (n=33, N=4)	p=0.2512	p<0.0001****	p<0.0001****
Lipofectamine (1:1) only (n=88, N=2)	p=0.0004***	p<0.0001****	p=0.1440

Supplementary Table S6. p-values of 7-11 DIV neurons treated with only niosomes or naked plasmids compared with untreated neurons in MTT assays. Red p-values mean that there is statistical difference with groups with higher mean values than the untreated groups, while black p-values mean that there is statistical difference with groups with lower mean values than the untreated groups.

Niosome	Size (nm)	Zeta Potential (mV)	PDI
ND12	89 ±1.3	-23± 0,3	0.21±0.08
P10	123.5±12.5	37.0 ±7.6	0,35±0.07
CQ	118.2±1.5	28.9±.7.8	0.13±0.02

Supplementary Table S7. Physicochemical characterization of niosome formulations in terms of size, zeta potential, and polydispersity index. Each value represents the mean ± SD of three measurements.
